# Adjuvant endocrine therapy with cyclin-dependent kinase 4/6 inhibitor, ribociclib, for localized hormone receptor-positive/HER2– breast cancer (LEADER)

**DOI:** 10.1038/s41523-024-00708-5

**Published:** 2025-01-07

**Authors:** Laura M. Spring, Lauren Scarpetti, Arielle J. Medford, Andrzej Niemierko, Amy Comander, Therese Mulvey, Lowell Schnipper, Steven J. Isakoff, Beverly Moy, Seth A. Wander, Jennifer Shin, Zanta Ephrem, Anneke R. Laposta, Elyssa Denault, Elizabeth Abraham, Gayle Calistro, Ekaterina Kalashnikova, Angel Rodriguez, Minetta C. Liu, Alexey Aleshin, Jeffrey Peppercorn, Leif W. Ellisen, Aditya Bardia

**Affiliations:** 1 Massachusetts General Hospital Cancer Center, Harvard Medical School, Boston, MA USA; 2 Beth Israel Deaconess Medical Center, Harvard Medical School, Boston, MA USA; 3 Natera Inc., Austin, TX USA; 4 Present Address: University of California Los Angeles, Los Angeles, USA

**Keywords:** Breast cancer, Breast cancer, Tumour biomarkers, Targeted therapies, Targeted therapies

## Abstract

Optimal timing and dosing of adjuvant cyclin-dependent kinase (CDK) 4/6 inhibitor in early breast cancer is controversial. This prospective phase II clinical trial investigated tolerability and safety of two ribociclib dosing schedules. Patients with stage I–III hormone receptor-positive (HR+)/HER2– breast cancer on adjuvant endocrine therapy (ET) were randomized to two ribociclib dosing schedules: 400 mg continuous vs 600 mg intermittent, with initiation in early (prior ET < 2 years) vs delayed (prior ET ≥ 2 years) setting. Primary objective was to evaluate safety and tolerability of continuous vs intermittent schedule. Primary endpoint was proportion of patients who discontinued ribociclib before completion of all 12 cycles (measured at 12 months). Recurrence free survival (RFS) and circulating tumor DNA (ctDNA) detection were also evaluated. 81 patients were enrolled. Only six serious adverse events occurred, with no significant difference between treatment arms and no subject deaths. Twenty-five patients (31%) discontinued ribociclib before completion of 12 months, with no significant difference between treatment arms. Ribociclib discontinuation was higher in early vs delayed initiation (36% vs 21%). At median follow-up of 20 months, two patients in the intermittent arm (600 mg; Arm 2) experienced disease recurrence (2-year RFS 97%, 95%CI 88–99%), vs none in the continuous arm (400 mg; Arm 1) (2-year RFS 100%). ctDNA was only identified in the two subjects with recurrent disease at median of 7.5 months prior to radiological recurrence. Ribociclib is a safe and well-tolerated adjunct to adjuvant ET in early-stage breast cancer. Delayed initiation of ribociclib at 400 mg continuous dosing was feasible, better tolerated and associated with promising outcomes. ctDNA detection preceded clinical evidence of recurrence and may be considered as a surveillance tool in breast cancer.

## Introduction

The majority of breast cancers are diagnosed at an early stage of disease^1,2^. For hormone receptor-positive (HR+) breast cancer, endocrine therapy (ET) is a key component of systemic therapy^3,4^. As such, ET is a standard adjuvant treatment for HR+, HER2– early-stage breast cancer. In this setting, ET has demonstrated significant reduction in recurrence risk and death^5,6^. Despite the standard 5-year duration of ET, the absolute risk of distant recurrence of early-stage breast cancer within 15 years after completion of therapy ranges from 10 to 17%^7^. The risk of recurrence is substantially greater for patients with high-risk clinicopathologic features, particularly during the first few years on adjuvant ET^8^.

Given the substantial recurrence risk associated with high risk early-stage breast cancer even with ET, there is considerable interest in combination therapy, particularly cyclin-dependent kinase (CDK) 4/6 inhibitors for patients with high-risk disease. In the metastatic setting, ET combined with CDK 4/6 inhibitor has demonstrated a significant improvement in clinical outcomes, with ribociclib and abemaciclib significantly improving overall survival^9–21^. Three drugs (ribociclib, palbociclib, and abemaciclib) have been approved for the treatment of HR+/HER2– advanced-stage breast cancer in combination with ET. There has been considerable interest in evaluation of CDK 4/6 inhibitors in early-stage disease. However, two phase III studies exploring palbociclib have reported negative results^22–24^, while a phase III study investigating abemaciclib for patients with high-risk, node-positive disease reported positive results^25,26^. Recently, an abstract announced the phase III NATALEE study exploring three years of ribociclib demonstrated statistically significant improvement in invasive disease-free survival in patients with stage II and III HR+/HER2– breast cancer, including those with no nodal involvement^27^. These large phase III adjuvant clinical trials have evaluated early use of CDK 4/6 inhibitors (within 24 months of surgery). As such, the role of delayed use of CDK 4/6 inhibitor (after 24 months of surgery) remains unknown. Given the biological phenomenon of cellular dormancy in HR+ breast cancer^28,29^, the timing of drugs that block proliferating cells, such as CDK 4/6 inhibitors, might be particularly relevant in this setting. Furthermore, while MONALEESA trials investigated 600 mg of ribociclib, NATALEE trial evaluated 400 mg, though direct comparison of 600 mg vs 400 mg in adjuvant setting remains unknown.

To address the unmet need, we conducted a prospective phase II clinical trial (LEADER) which investigated the use of ribociclib in two different dosing schedules (600 mg vs 400 mg) and time of initiation (early, prior ET < 2 years vs delayed, prior ET ≥ 2 years) initiation with standard adjuvant ET for patients with HR+/HER2– early-stage breast cancer. The goal of the trial was to investigate and compare tolerability and disease recurrence of two ribociclib dosing schedules. Additionally, this trial evaluated the relationship between ctDNA detection and recurrence-free survival (RFS).

## Results

### Patient Characteristics

A total of 81 patients were enrolled in Cohort 1 between February 2, 2018 and September 27, 2019: 41 patients on continuous schedule (51%) and 40 patients on intermittent schedule (49%). One patient was lost to follow-up, while two patients withdrew from the trial prior to initiating treatment. Patient characteristics are shown in Table 1. Median age at randomization was 54 years (range 34–75), and all patients enrolled were female. Of these women, 37% were pre- or perimenopausal and 63% were postmenopausal at the time of study enrollment. The majority of patients were treated with chemotherapy prior to trial enrollment (77%) in either the neoadjuvant or adjuvant setting. All premenopausal patients were concurrently treated with a luteinizing hormone-releasing hormone (LHRH) agonist during the study. The only significant difference observed in baseline characteristics between treatment arms was type of initial adjuvant ET (*p* = 0.04). A greater number of patients were treated with ET for > 5 years prior to starting ribociclib on the continuous arm (400 mg; Arm 1) compared to the intermittent arm (600 mg; Arm 2); however, this difference was not statistically significant (*p* = 0.06).

**Table 1:** Patient characteristics in the clinical trial

	Total (*n* = 81)	Continuous (Arm 1; *n* = 41)	Intermittent (Arm 2; *n* = 40)	*p* value
**Age at randomization, years (range)**	54 (34–75)	53 (49–62)	55 (47–61)	0.53
**Age group** **≤** **50**	27 (33%)	12 (29%)	15 (38%)	0.43
**Sex**				
Female	81 (100%)	41 (100%)	40 (100%)	
Male	0 (0%)	0 (0%)	0 (0%)	
**Menopausal status**				0.59
Pre- or perimenopausal	30 (37%)	14 (34%)	16 (40%)	
Postmenopausal	51 (63%)	27 (66%)	24 (60%)	
**Race**				0.32
Asian	4 (5%)	3 (7%)	1 (2%)	
Black or African American	3 (4%)	0 (0%)	3 (8%)	
More than one race	3 (4%)	1 (2%)	2 (5%)	
Other or unknown	7 (9%)	3 (7%)	4 (10%)	
White	64 (79%)	34 (83%)	30 (75%)	
**Ethnicity**				0.24
Hispanic or Latino	5 (6%)	2 (5%)	3 (8%)	
Non-Hispanic	63 (78%)	35 (85%)	28 (70%)	
Unknown	13 (16%)	4 (10%)	9 (22%)	
**Disease stage**				0.93
I	11 (14%)	6 (15%)	5 (12%)	
II	45 (56%)	23 (56%)	22 (55%)	
III	25 (31%)	12 (29%)	13 (32%)	
**Histological grade**				0.23
1	11 (14%)	8 (20%)	3 (8%)	
2	46 (57%)	23 (56%)	23 (57%)	
3	24 (30%)	10 (24%)	14 (35%)	
**Estrogen receptor-positive**	81 (100%)	41 (100%)	40 (100%)	
**Progesterone receptor status**				0.25
Low positive	1 (1%)	1 (2%)	0 (0%)	
Positive	71 (88%)	37 (90%)	34 (85%)	
Negative	8 (10%)	2 (5%)	6 (15%)	
Unknown	1 (1%)	1 (2%)	0 (0%)	
**Surgery**				0.71
Bilateral mastectomy	26 (32%)	13 (32%)	13 (32%)	
Unilateral mastectomy	29 (36%)	14 (34%)	15 (38%)	
Lumpectomy or conservation	25 (31%)	14 (34%)	11 (28%)	
None	1 (1%)	0 (0%)	1 (2%)	
**Radiotherapy**				0.97
Adjuvant	71 (88%)	36 (88%)	35 (88%)	
None	10 (12%)	5 (12%)	5 (12%)	
**Chemotherapy (general)**				0.75
Adjuvant or neoadjuvant	62 (77%)	32 (78%)	30 (75%)	
None	19 (23%)	9 (22%)	10 (25%)	
**Anthracycline therapy**				0.32
Yes	46 (57%)	23 (56%)	23 (57%)	
No	33 (41%)	18 (44%)	15 (38%)	
Unknown	2 (2%)	0 (0%)	2 (5%)	
**Taxane therapy**				0.56
Yes	61 (75%)	32 (78%)	29 (72%)	
No	20 (25%)	9 (22%)	11 (28%)	
**Initial adjuvant endocrine therapy**				0.04
Aromatase inhibitor	63 (78%)	28 (68%)	35 (88%)	
Tamoxifen	18 (22%)	13 (32%)	5 (12%)	
**Concurrent adjuvant LHRH agonist**				0.59
Yes	30 (37%)	14 (34%)	16 (40%)	
No	51 (63%)	27 (66%)	24 (60%)	
**Duration of endocrine therapy (prior to randomization)**				0.06
< 2 years	53 (65%)	23 (56%)	30 (75%)	
2–5 years	20 (25%)	11 (27%)	9 (22%)	
> 5 years	8 (10%)	7 (17%)	1 (2%)	

### Safety (adverse events)

Observed AEs are listed in Supplemental Table 1, most of which were hematologic. Overall, the most common grade 3 or 4 toxicities (≥10%) were neutropenia (experienced in 35% of subjects), lymphocytopenia (12% of subjects), and leukocytopenia (11% of subjects). Grade 3 or 4 neutropenia occurred in 24% of subjects on the continuous arm (Arm 1) and 45% of subjects on the intermittent arm (Arm 2). There were twelve instances total of QTcF prolongation (83% grade 1, 8% grade 2, 8% grade 3), with no significant difference between study arms (*p* = 0.49). Grade 1 constipation was significantly more common among patients on the continuous arm (Arm 1) compared to those on the intermittent arm (Arm 2) (41% vs 20%, respectively; *p* = 0.04). Overall, six serious adverse events (SAEs) occurred during the trial among four subjects (one on the continuous arm (Arm 1), three on the intermittent arm (Arm 2): lymphocytopenia, elevated alanine aminotransferase, elevated aspartate aminotransferase, skin infection, soft tissue infection, and obstruction of the small intestine. There was no significant difference in the proportion of SAEs between treatment arms (*p* = 0.4).

### Therapy adherence

**Fig. 1:**
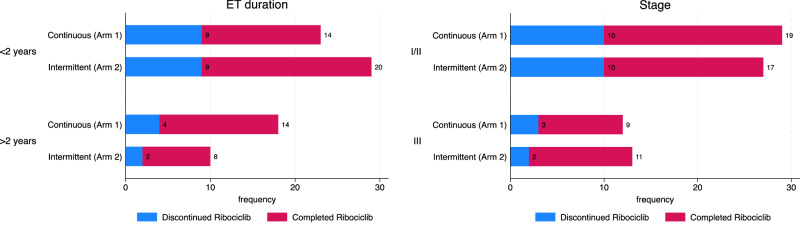
Treatment Discontinuation Rate. **A**, **B** Proportion of patients who discontinued CDK 4/6 treatment before the completion of 12 months, stratified by prior duration of ET < 2 years vs ≥ 2 years (**a**), stage I/II vs III (**b**).

Twenty-five patients (31%) discontinued ribociclib treatment before completion of 12 months, with no significant difference between intermittent versus continuous schedule. Thirteen patients discontinued ribociclib early on the continuous arm (Arm 1), while 12 patients discontinued early on the intermittent arm (Arm 2) (*p* = 0.9). The ribociclib discontinuation rate for patients was numerically higher among patients on ET for < 2 years vs ≥ 2 years (36% vs 21%, respectively), as outlined in Fig. 1a. Ribociclib discontinuation was not associated with other factors including age, stage, menopausal status, or prior chemotherapy (Fig. 1b, Supplemental Fig. 2 and Table 2).

**Fig. 2:**
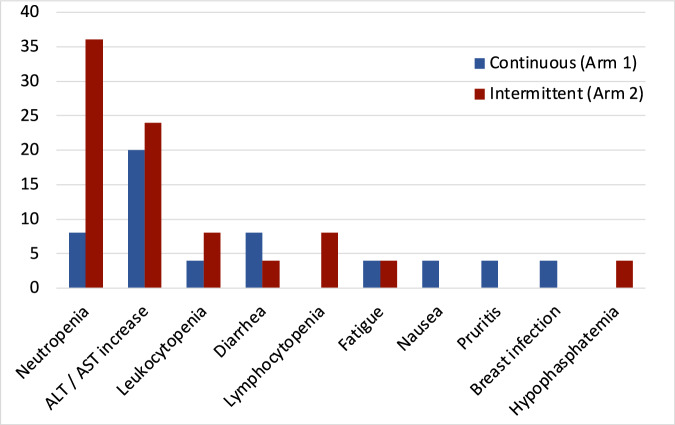
Most common adverse events leading to treatment discontinuation. The most common adverse events leading to treatment discontinuation are listed stratified by ribociclib dosing (continuous versus intermittent). The continous arm is highlighted in blue and intermittent in red.

Early discontinuation (discontinuation prior to completion of 12 months of ribociclib therapy) was attributed to unacceptable adverse events (15 subjects; 60%), voluntary treatment withdrawal (8 subjects; 32%), patient relocation (1 subject; 4%), and intercurrent illness (1 subject; 4%). The most common grade 3 or greater adverse events (AEs) leading to study discontinuation are shown in Fig. 2. Among patients who discontinued early, neutropenia was more frequent in the intermittent arm (Arm 2), 9 of 12 patients (75%), versus 2 of 13 patients (15%) in the continuous arm (Arm 1). No patients discontinued early due to prolonged QTc. Ribociclib was dose reduced for eight patients (20%) on the continuous arm (Arm 1), and for fourteen (35%) on the intermittent arm (Arm 2) (Fig. 2).

### Biomarker outcomes

**Fig. 3:**
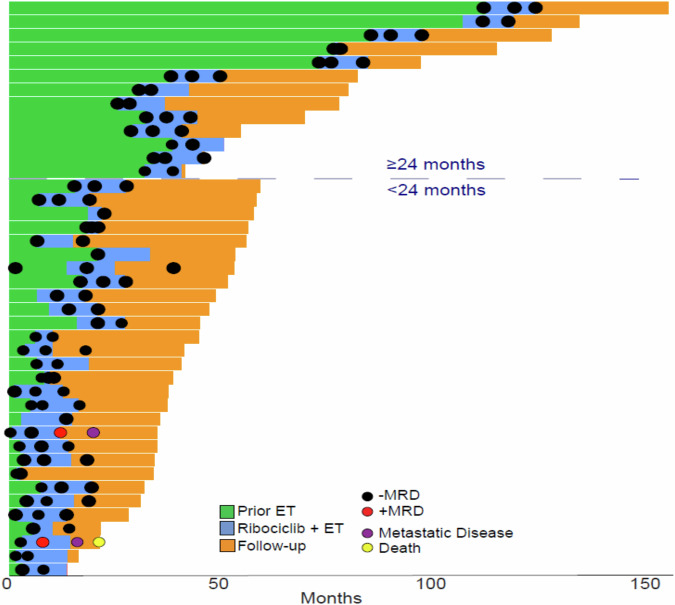
Swimmer plot showing patients’ disease courses, including endocrine therapy prior to trial enrollment (green), up to 12 months on combined ribociclib and endocrine therapy on the LEADER clinical protocol (blue), and follow-up after ribociclib completion (orange). Black circles indicate negative MRD test, and red circles indicated positive MRD test. Purple circles indicate metastatic recurrence, and yellow circle indicates death.

Of the 81 patients enrolled, 42 patients (52%) had ctDNA collected and successfully retrospectively analyzed for at least one timepoint (Fig. 3). The remaining 39 patients did not have samples suitable for ctDNA analysis due to the following limitations: missing tumor tissue (*n* = 18), tumor tissue failed pathology (*n* = 10), missing matched normal samples (*n* = 2), discordant tumor/normal whole exome sequencing (*n* = 2), and samples not available for analysis (*n* = 7). There were no statistically significant differences in baseline characteristics observed between patients who had ctDNA collected and successfully retrospectively analyzed for at least one timepoint versus those who did not (Supplemental Table 3). Median follow-up period between these two patient groups was also comparable (19.7 months versus 20.1 months, respectively).

**Fig. 4:**
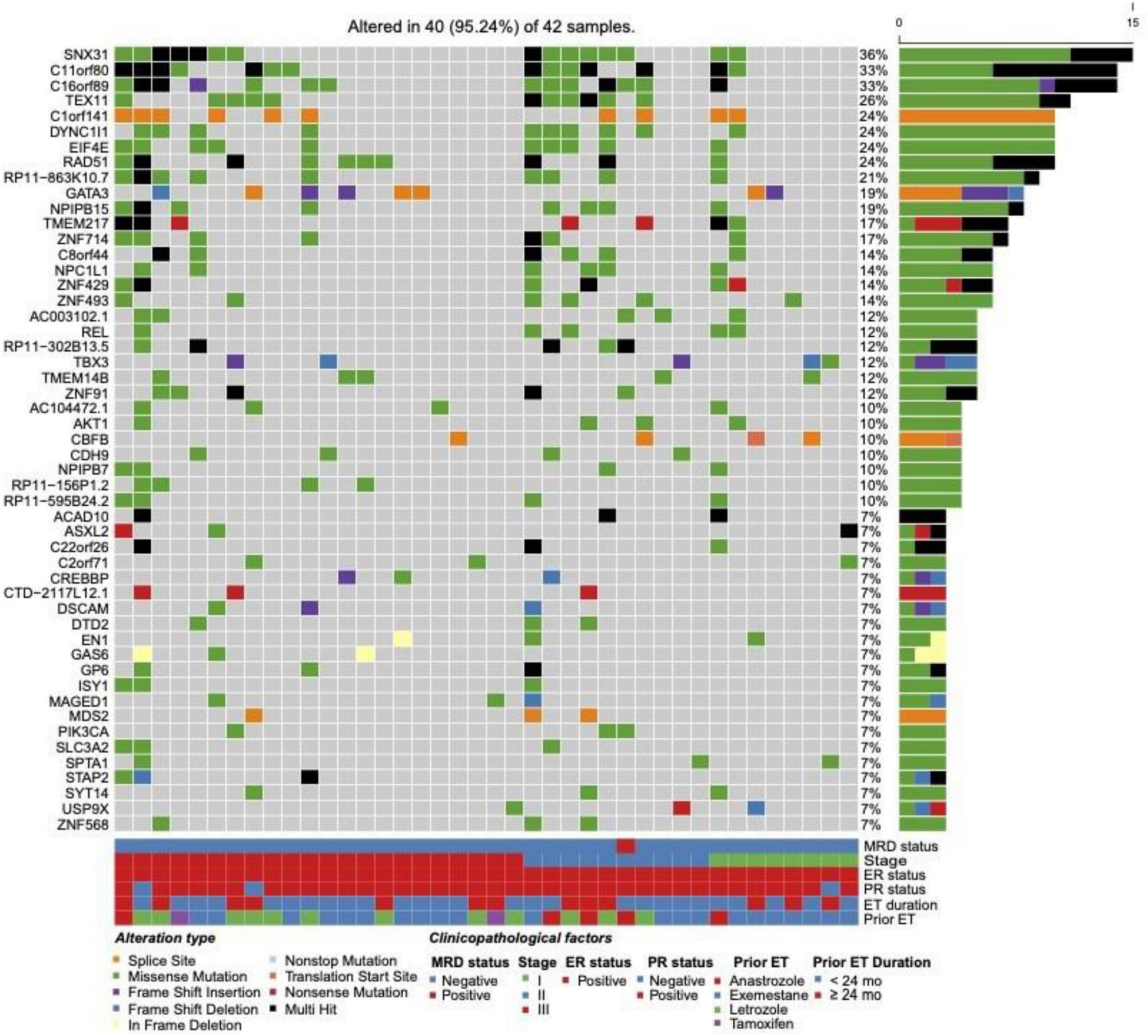
Comutation plot depicting commonly altered genes in 40/42 (95%) samples that underwent initial next generation sequencing. Notably, one patient with MRD did not have significantly overlapping genomic variants and thus is not listed in the above plot. Additional clinical data includes minimal residual disease status, stage, hormone receptor status, prior endocrine therapy duration, and identity of prior endocrine therapy. MRD minimal residual disease, ER estrogen receptor; PR progesterone receptor; ET endocrine therapy.

Among the analyzed samples, two samples from two different subjects were positive for ctDNA (4.8% of patients (*n* = 42); 1.75% of samples (*n* = 103)). These two patients identified as positive for ctDNA were the only patients to develop clinical evidence of recurrent disease, as detailed below. Figure 4 depicts the commonly altered genes in 40/42 (95%) samples that underwent initial next generation sequencing; of note, one patient with MRD did not have significantly overlapping genomic variants and is not among the 40 patients. Genomic alterations were noted in *SNX31*, *RAD51*, *GATA3*, *AKT1*, *PIK3CA*, and *ZNF* family, at different variant allelic frequencies.

### Survival outcomes

No patients had detectable ctDNA at screening and/or Cycle 1 Day 1 of treatment. After a median follow-up of 20 months, two patients, both in the intermittent arm (600 mg; Arm 2), experienced disease recurrence. Both of these patients had positive ctDNA detected prior to clinical evidence of recurrence, one patient 7 months and the other 8 months prior to clinical progression (Supplemental Fig. 3).

**Fig. 5:**
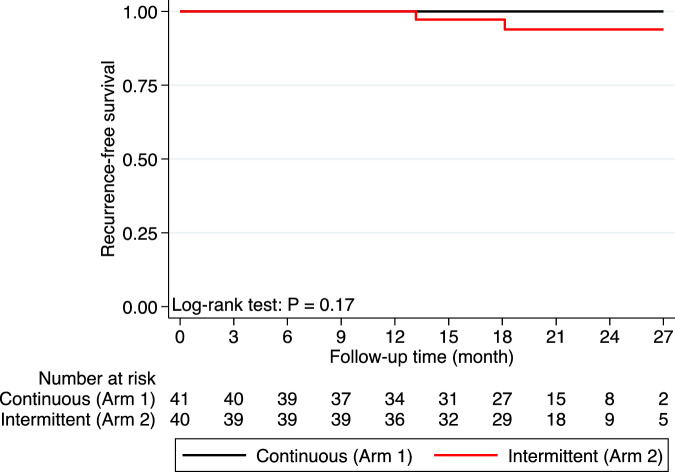
Kaplan–Meier curve of recurrence-free survival with ribociclib.

In one patient, ctDNA was detectable after 5 months on ribociclib, and for the other ctDNA was detectable immediately upon completion of 12 months ribociclib. One patient developed brain-only metastases. Notably, neither patient had undergone radiographic evaluation of the sites of metastasis until clinical evidence of metastatic disease. Subsequent targeted ctDNA testing in the patient with visceral metastases 4 months after metastatic diagnosis identified high level *CCND1* amplification, medium *EGFR* amplification, and low level *CCNE1* amplification, as well as a *TP53* L194R mutation (mutate allele fraction 25.1%). Overall, RFS was 100% at 1 year and 97% (95% CI 88–99%) at 2 years. At two years, RFS was 100% for the continuous arm (Arm 1) and 94% (95% CI 78– 98%) for the intermittent arm (Arm 2) (Fig. 5).

## Discussion

To our knowledge, LEADER is the first prospective clinical trial to directly investigate a) 600 mg intermittent vs 400 mg continuous ribociclib and b) early vs delayed start of ribociclib in combination with adjuvant ET for patients with localized HR+/HER2– breast cancer, with a duration of 12 months. While ribociclib was well-tolerated among both arms, AEs were higher with 600 mg than 400 mg. In total, 31% of patients discontinued ribocolib treatment before completion of 12 months of therapy (32% of patients on the continuous arm vs 30% on the intermittent arm). Early discontinuation did not significantly differ between treatment arms. More patients treated with ET < 2 years had early discontinuation compared to patients treated with ET ≥ 2 years prior to study enrollment, which could have therapeutic implications for dosing and timing of CDK 4/6 inhibitor use. At a median follow-up period of 20 months, none of the patients in the continuous arm (400 mg; Arm 1) had disease recurrence (RFS at 2 years of 100%).

The early discontinuation rate is similar to that of other trials comparing adjuvant ET plus CDK 4/6 inhibitor versus adjuvant ET with placebo^22,23,25,26^. AEs were the major reason for early discontinuation of ribociclib therapy. Among patients who discontinued early, neutropenia was more frequent in the intermittent arm (600 mg; Arm 2) than the continuous arm (400 mg; Arm 1). Discontinuation did not differ significantly between patients who had received prior chemotherapy versus those who had not, which demonstrates that ribociclib can be safely administered as an adjunctive treatment to current standard of care. Ribociclib tolerability was not affected by age and/or menopausal status.

Overall, ribociclib discontinuation was less common in the delayed setting. This suggests that ribociclib may be better tolerated after prolonged ET. Patients who have been on ET for a longer duration may tolerate ET better than those who are first starting, which may increase the odds of tolerating the addition of a CDK 4/6 inhibitor. Prior studies and this trial suggest safety of CDK 4/6 inhibitors at any point in the adjuvant setting, but these drugs may be better tolerated in a delayed setting by allowing patients more time to recover from traditional antiproliferative therapies prior to use. A substantial number of patients decided to withdraw from the trial voluntarily due to treatment side effects (though not determined to be unacceptable AEs per protocol) and/or unwillingness to participate in trial requirements. It is possible that patients who have been on breast cancer therapies for a longer duration of time are more accustomed to treatment-related side effects and/or increased interventions. This is particularly important because most of the ongoing (and completed) adjuvant trials are examining the use of a CDK 4/6 inhibitor in localized disease early in the adjuvant setting. Future research is needed to build on these observations and identify optimal timing for CDK 4/6 inhibitors in the adjuvant setting.

Analysis of ctDNA using a tumor-informed assay revealed the two patients who experienced disease recurrence thus far were the only two patients with detectable ctDNA. The detectable ctDNA levels were very low at 0.1 MTM/mL and in one patient and 0.2 MTM/mL in the other. Detection of these very low levels of ctDNA in the adjuvant setting highlights the need for better, ultra-sensitive assays in the adjuvant setting, especially as previous research has already shown that ctDNA can reliably predict relapse in early-stage breast cancer^30,31^. In this study, our ultra-sensitive ctDNA assay was sufficient to detect recurrence 7 and 8 months prior to clinical evidence. Notably, the initial time points for both patients were negative. Furthermore, one patient experienced recurrence in the brain only, which is often considered a metastatic site less likely to shed ctDNA into the plasma, due to the blood brain barrier^32,33^. Also notable was the fact that one patient had detectable ctDNA while on ribociclib and another immediately upon treatment completion. These findings question whether ctDNA detection calls for a change in treatment, a clinical question that is undergoing further investigation in clinical trials (DARE NCT04567420, TRAK-ER NCT04985266). Similarly, subsequent ctDNA analysis after metastatic diagnosis identified one patient with recurrence had, among other alterations, low level amplification of *CCNE1*, which has been described as a potential CDK resistance mechanism^34,35^ potentially contributing to recurrence.

Overall, only two patients had recurrent disease since completion of ribociclib treatment, both of whom were treated with 600 mg intermittent schedule. It is interesting that recurrence only occurred among patients treated with intermittent ribociclib therapy, but small numbers preclude definitive conclusions. CDK 4/6 inhibitors are known to promote tumor suppression by inducing cellular transition to quiescence or senescence^36,37^. Through the induction of senescence, CDK 4/6 inhibitors irreversibly inhibit tumor cell growth and promote immune-mediated clearance^36^. Continuous dosing of CDK 4/6 inhibitor likely allows for greater efficacy in cytotoxic T cell recruitment and tumor destruction. For cells that undergo quiescence, growth arrest is reversible, and cancer cells will immediately re-enter the cell cycle at withdrawal of CDK 4/6 inhibition^38^. However, these findings are hypothesis generating and further validation is required.

It is encouraging to note the low risk of disease recurrence overall. Recent randomized trials have shown that CDK 4/6 inhibition combined with ET reduces the risk of disease recurrence. One of such trials was monarchE, which demonstrated that abemaciclib plus standard ET yielded superior invasive disease-free survival and distant recurrence-free survival compared to ET alone^25,26^. This led to the recent FDA approval of abemaciclib for the adjuvant treatment of HR+/HER2–, high risk early breast cancer^39,40^. However, the AE profile with abemaciclib is different than ribociclib with diarrhea being more common with former and neutropenia more common with latter^39^. The phase III NATALEE study looking at 3 years of ribociclib 400 mg was recently shown to significantly improve invasive disease–free survival and demonstrated a favorable safety profile^27^. As such, it is reasonable to consider ribociclib for adjuvant treatment of stage II or III HR+/HER2− early-stage breast cancer, and it is anticipated that FDA approval may also extend to ribociclib in the near future.

This study has few limitations, most notably the limited follow-up period and modest sample size. There was a significant difference in initial ET used among the two treatment arms. However, this is unlikely to affect study results. It should be noted that numerically more patients in the continuous arm (Arm 1) had ET for > 5 years prior to study treatment, but this difference did not meet statistical significance. While two different ribociclib doses were studied, there was no control group receiving ET alone, and this study is underpowered to evaluate disease-free survival. In addition, approximately half of study patients had ctDNA samples successfully collected and analyzed, which is indicative of the difficulty in sample collection for tumor-informed assays and the need for more feasible methods for capturing ctDNA.

In summary, this study contributes to emerging evidence supporting the use of ribociclib in early-stage HR+/HER2– breast cancer. Although this trial did not directly assess the impact of therapeutic schedule and timing in modulating response to CDK 4/6 inhibitors in early breast cancer, the study findings indicate that this should be more thoroughly explored. The majority of patients tolerated one-year of ribociclib therapy regardless of dosing schedule. However, this study provides a potential signal that ribociclib at lower dose (400 mg) on a continuous schedule may be better tolerated and may cause less toxicity than higher dose (600 mg) over an intermittent schedule. The study also evaluates the potential role of ultra-sensitive, personalized ctDNA detection in the adjuvant breast cancer setting for disease recurrence monitoring and requires validation in future studies. Overall, ribociclib is a promising adjunctive treatment to standard adjuvant ET regimens for early-stage breast cancer.

## Methods

LEADER is a multicenter, randomized, phase II clinical trial investigating two different schedules (intermittent vs continuous) of ribociclib in combination with adjuvant ET for patients with localized HR+/HER2– breast cancer (NCT03285412; registered September 18, 2017). Cohort 1 of the LEADER trial (presented in this manuscript) included patients with early-stage disease with at least one more year of planned adjuvant ET remaining, stratified by prior duration of ET (less than 2 years vs 2 or more years). Cohort 2 of LEADER (ongoing) focuses on subjects with positive ctDNA.

### Patient population

Pre- and postmenopausal women and men ≥ 18 years of age with localized HR+/HER2– breast cancer of T1c-T4c, any N, M0, as per AJCC 8^th^ edition. Patients could enroll within any ET duration in the adjuvant setting as long as there was a plan for at least one more year of adjuvant ET (initiated prior to randomization). The protocol allowed for concurrent treatment with a gonadotropin-releasing hormone agonist for premenopausal women and required adequate bone marrow, liver, and renal function. Additional details included in supplementary section.

The trial was approved by the Dana Farber/Harvard Cancer Center Institutional Review Board. This research has complied with all relevant ethical regulations. The trial was conducted in accordance with the International Conference on Harmonization Good Clinical Practice Guidelines (ICH GCP) as well as the Declaration of Helsinki and was registered at ClinicalTrials.gov (NCT03285412). All patients provided written informed consent prior to initiation of any study-related treatment or procedures.

### Study design and treatment plan

Eligible patients were randomized (1:1) into two ribociclib treatment groups: 400 mg continuous (daily of 28-day cycle; Arm 1) or 600 mg intermittent (days 1–21 of 28–day cycle; Arm 2) for 12 months. Randomization was stratified according to duration of endocrine therapy ( < or ≥ 5 years) and whether patient received prior chemotherapy. Ribociclib was taken orally, once daily at the same time each day (±4 hours). Patients were concurrently treated with standard adjuvant ET (letrozole, anastrozole, or exemestane) plus a luteinizing hormone-releasing hormone (LHRH) agonist in premenopausal patients, which was initiated prior to randomization. There was no maximum or minimum required duration of ET prior to study enrollment.

### Safety and efficacy assessments

AEs were graded according to the National Cancer Institute Common Terminology Criteria for Adverse Events (CTCAE) version 4.03. Cardiac toxicity was monitored more intensively than recommended by the United Surgical Partners International (USPI), during Cycle 1, 2, 4, 6, and 12 by regular electrocardiographs (Cycle 1 Days 1, 15; Cycle 2 Days 1, 15; and Day 1 of Cycles 4, 6, and 12). Hematology and chemistry assessments were performed during Cycles 1, 2, 3 and every subsequent even cycle (Cycle 1 Days 1, 15; Cycle 2 Days 1, 15; Cycle 3 Day 1; and Day 1 of every subsequent even cycle). Information on toxicity management is included in the Supplementary Methods.

### Biomarker assessment

Blood samples were collected at screening, Cycle 1 Day 1, and Day 1 of every even cycle; samples were then pooled and sent for ctDNA via Signatera, a personalized, tumor-informed assay^41^. Briefly, whole exome sequencing (WES) was performed on formalin fixed and paraffin embedded (FFPE) tumor tissues along with matched normal blood samples. A set of 16 high-ranked, patient-specific, somatic, clonal single nucleotide variants (SNVs) were selected for multiplex (m)PCR testing. The mPCR primers targeting the personalized SNVs were designed and synthesized to track ctDNA in patients’ plasma. Plasma samples were retrospectively analyzed and samples were considered ctDNA-positive when SNVs detected. ctDNA concentration was reported as mean tumor molecules (MTM)/mL of plasma. ctDNA analyzers were blinded to the clinical results.

### Outcomes

The primary objective of Part I of LEADER was to evaluate the safety and tolerability of intermittent vs continuous schedule of ribociclib in combination with ET. The primary endpoint was the proportion of patients who discontinued ribociclib before the completion of all 12 cycles. Key secondary endpoints included adherences stratified by early (prior ET < 2 years) versus delayed (prior ET ≥ 2 years) initiation of adjuvant ribociclib, RFS and detection of ctDNA. Recurrence-free interval was defined as the time of registration to event, including local or regional relapse, distant relapse, or death from breast cancer. All patients who received at least one dose of protocol therapy were evaluated for clinical benefit. Follow-up assessments (physical examination, AE evaluation, ctDNA collection, questionnaires) were performed every 6–12 months for three years after end of treatment.

### Statistical analysis

The primary statistical objective of Part 1 is to provide inference and confidence interval for a single proportion (66%, percentage of patients on each arm who can complete 12 months of therapy, as the primary trial objective was the evaluation of safety and tolerability of study treatment. Given the data on endocrine monotherapy compliance, a 45% or lower proportion of ribociclib treatment will be a null hypothesis^42^. Accordingly, a sample size of ~ 80 patients (40 in each arm) will provide 80% power to discriminate between each treatment arm and the historical null hypothesis with a two-sided test at 0.05 significance level (p0 = 0.45; p1 = 0.66). All descriptive and actuarial analyses were performed using STATA (StataCorp. 2021. Stata Statistical Software: Release 17. College Station, TX: StataCorp LLC). Comparisons between groups were done using Wilcoxon rank-sum test for continuous variables and Pearson’s chi-squared test for categorical and binary variables. Kaplan-Meier methodology was used to estimate RFS. RFS was defined as time period since randomization date. The RFS curves were compared using the log-rank test. Median follow-up period was defined as time period since randomization date. A p-value less than 0.05 was considered to be statistically significant. Descriptive statistics were used for ctDNA analysis as these tests were exploratory.

## Supplementary information

Supplementary Material

Supplementary DNA Sequencing Data

## Data Availability

In order to be compliant with the ethics committee and to protect the privacy and confidentiality of patients in this study, the sequencing data and source data supporting the findings of this study are not made publicly available but can be requested from the corresponding author (ABardia@mednet.ucla.edu) for academic use only, within the limitations of the provided informed consent. Data will not be made available for commercial use. Any request will be reviewed within a timeframe of 2-4 weeks to verify whether the request is subject to any intellectual property or confidentiality obligations. All data shared will be de-identified and will be provided to researchers with access limited for scientific verification purposes and with strict prohibitions on secondary use. Applying researchers will be required to sign a data usage agreement.

## Code availability

Code used for this study is available upon request from the corresponding author.
