# Supplementary material for: Adjuvant endocrine therapy with cyclin-dependent kinase 4/6 inhibitor, ribociclib, for localized hormone receptor-positive/HER2– breast cancer (LEADER)
Source: NPJ Breast Cancer. 2025 Jan 7;11:2. doi: 10.1038/s41523-024-00708-5 (PMC11707077; doi:10.1038/s41523-024-00708-5)
Supplement: Supplementary file 1 — Supplementary Material [file 41523_2024_708_MOESM1_ESM.pdf]

Supplementary Figures and Tables

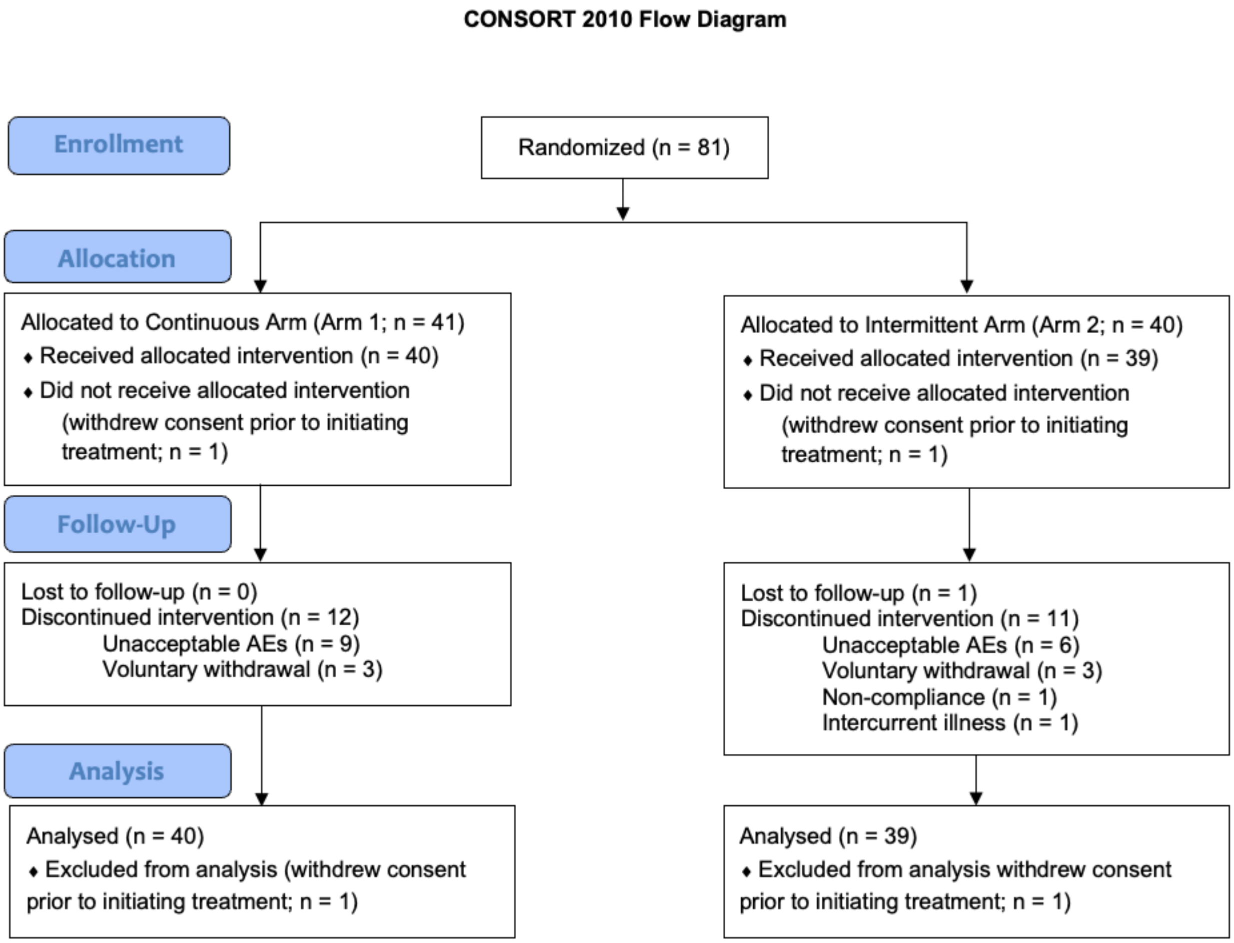

Supplemental Figure 1A. Consort diagram of patient enrollment in the clinical trial.

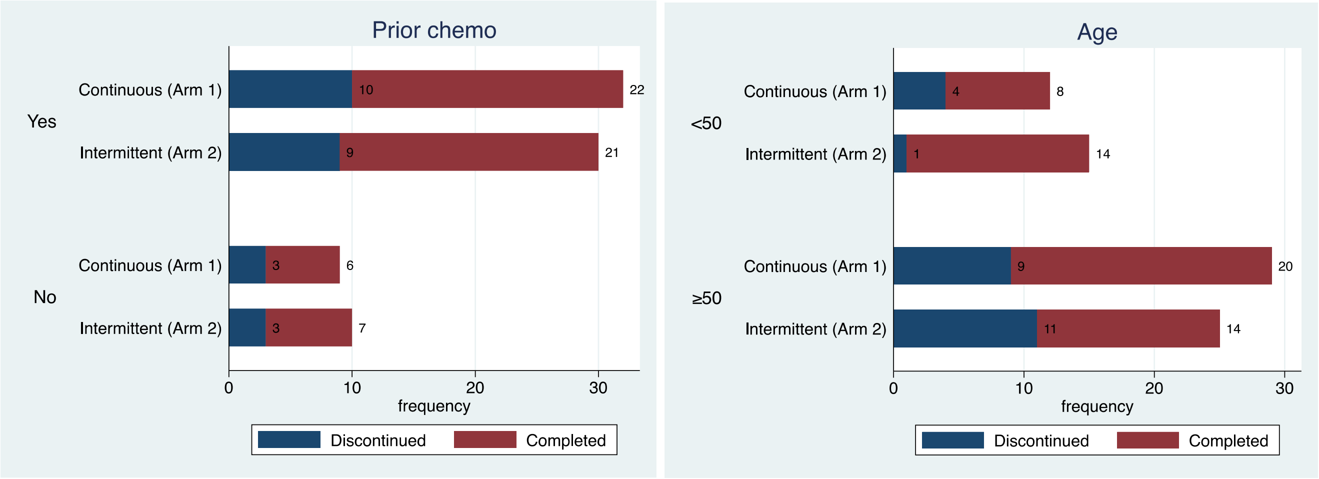

Supplemental Figure 2A and 2B. Proportion of patients who discontinued CDK 4/6 treatment before the completion of 12 months, stratified by prior chemotherapy (1a), age (1b).

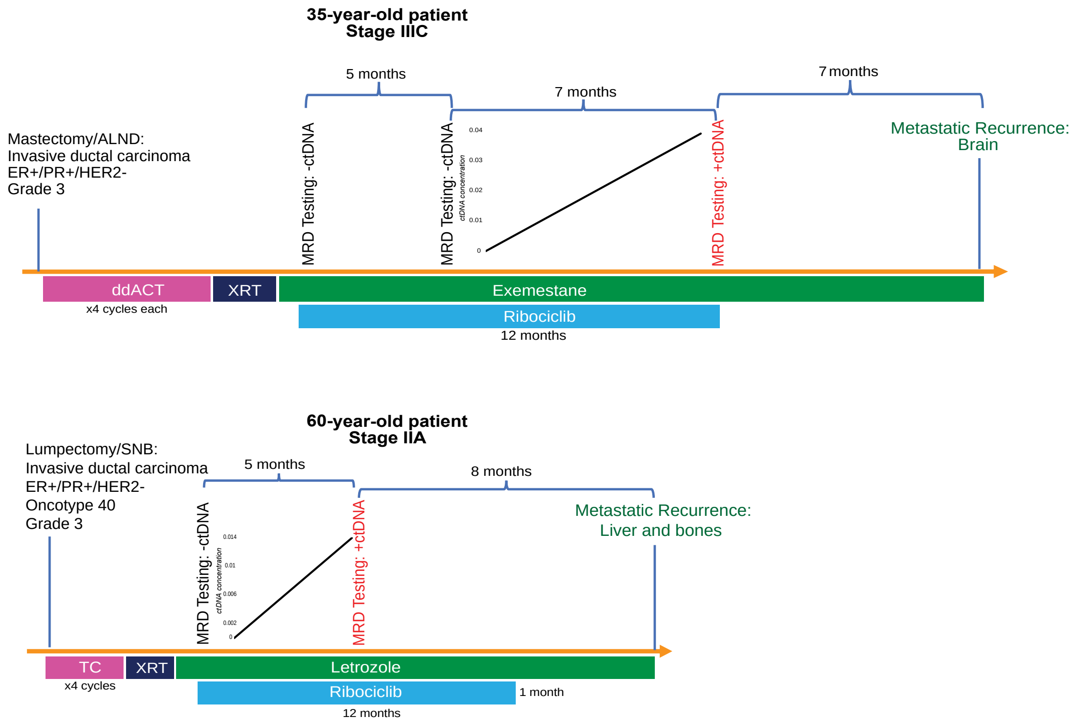

Supplemental Figure 3. Treatment timelines of the two patient who had positive minimal residual disease (MRD) tests. Treatments are indicated below the timeline, and MRD testing results are indicated with clinical data above the timeline. MRD results are reported at mean tumor molecules (MTM)/mL. ddACT = dose dense doxorubicin & cyclophosphamide followed by paclitaxel; TC = docetaxel & cyclophosphamide; XRT = adjuvant radiation.

Supplemental Table 1. Comprehensive list of adverse events seen with ribociclib in both arms.

Adverse Event		Continuous (Arm 1; n = 41)	Intermittent (Arm 2; n = 40)	p-value
**Wound infection**				0.37
	Grade 2	0 (0%)	1 (2%)	
	Grade 3	1 (2%)	0 (0%)	
**White blood cell decrease**				0.87
	Grade 1	15 (37%)	13 (32%)	
	Grade 2	15 (37%)	17 (42%)	
	Grade 3	4 (10%)	5 (12%)	
**Wheezing**				0.31
	Grade 1	0 (0%)	1 (2%)	
**Weight loss**				0.31
	Grade 2	0 (0%)	1 (2%)	
**Weight gain**				0.31
	Grade 1	0 (0%)	1 (2%)	
**Watering eyes**				0.08
	Grade 1	3 (7%)	0 (0%)	
**Vomiting**				0.38
	Grade 1	4 (10%)	5 (12%)	
	Grade 2	4 (10%)	1 (2%)	
**Vertigo**				0.99
	Grade 1	1 (2%)	1 (2%)	
**Vaginal pain**				0.31
	Grade 1	0 (0%)	1 (2%)	
**Vaginal infection**				0.32
	Grade 2	1 (2%)	0 (0%)	
**Vaginal dryness**				0.12
	Grade 1	0 (0%)	3 (8%)	
	Grade 2	0 (0%)	1 (2%)	
**Urine discoloration**				0.31
	Grade 1	0 (0%)	1 (2%)	
**Urinary tract pain**				0.32
	Grade 1	1 (2%)	0 (0%)	
**Urinary tract infection**				0.13
	Grade 1	2 (5%)	0 (0%)	
	Grade 2	2 (5%)	0 (0%)	
**Urinary frequency**				0.15
	Grade 1	0 (0%)	2 (5%)	
**Upper respiratory infection**				0.37
	Grade 1	0 (0%)	1 (2%)	
	Grade 2	1 (2%)	0 (0%)	
**Tricuspid valve disease**				0.41
	Grade 1	13 (32%)	16 (40%)	

	Grade 2	0 (0%)	1 (2%)	
**Tremor**				0.31
	Grade 1	0 (0%)	1 (2%)	
**Tooth infection**				0.32
	Grade 3	1 (2%)	0 (0%)	
**Thrombotic thrombocytopenia**				0.31
	Grade 1	0 (0%)	1 (2%)	
**Surgical and medical process**				0.37
	Grade 1	1 (2%)	0 (0%)	
	Grade 2	1 (2%)	0 (0%)	
**Stomach pain**				0.32
	Grade 2	1 (2%)	0 (0%)	
**Spasticity**				0.16
	Grade 1	2 (5%)	0 (0%)	
**Sore throat**				0.97
	Grade 1	3 (7%)	3 (8%)	
**Soft tissue infection**				0.37
	Grade 2	1 (2%)	0 (0%)	
	Grade 3	1 (2%)	0 (0%)	
**Small intestinal obstruction**				0.31
	Grade 3	0 (0%)	1 (2%)	
**Sleep apnea**				0.31
	Grade 1	0 (0%)	1 (2%)	
**Skin/subcutaneous tissue disease**				0.41
	Grade 1	4 (10%)	2 (5%)	
**Skin infection**				0.39
	Grade 1	0 (0%)	1 (2%)	
	Grade 2	3 (7%)	1 (2%)	
	Grade 3	0 (0%)	1 (2%)	
**Skin hyperpigmentation**				0.15
	Grade 1	0 (0%)	2 (5%)	
**Sinusitis**				0.16
	Grade 2	2 (5%)	0 (0%)	
**Sinus tachycardia**				0.41
	Grade 1	4 (10%)	2 (5%)	
**Sinus pain**				0.35
	Grade 1	0 (0%)	1 (2%)	
	Grade 2	0 (0%)	1 (2%)	
**Sinus bradycardia**				0.62
	Grade 1	2 (5%)	3 (8%)	
**Seroma**				0.32
	Grade 1	1 (2%)	0 (0%)	
**Retinal vascular disorders**				0.32

Grade 3	1 (2%)	0 (0%)	
**Respiratory thoracic and mediastinal disorders**			0.37
Grade 1	1 (2%)	0 (0%)	
Grade 2	1 (2%)	0 (0%)	
**Renal colic**			0.31
Grade 2	0 (0%)	1 (2%)	
**Rash maculopapular**			0.57
Grade 1	5 (12%)	4 (10%)	
Grade 3	0 (0%)	1 (2%)	
**Rash acneiform**			0.99
Grade 1	1 (2%)	1 (2%)	
**Pulmonary valve disease**			0.07
Grade 1	7 (17%)	14 (35%)	
**Psychiatric disorders**			0.32
Grade 1	1 (2%)	0 (0%)	
**Pruritus**			0.80
Grade 1	4 (10%)	4 (10%)	
Grade 2	1 (2%)	1 (2%)	
Grade 3	1 (2%)	0 (0%)	
**Proteinuria**			0.32
Grade 1	1 (2%)	0 (0%)	
**Productive cough**			0.31
Grade 1	0 (0%)	1 (2%)	
**Postnasal drip**			0.16
Grade 1	2 (5%)	0 (0%)	
**Pneumonitis**			0.99
Grade 2	1 (2%)	1 (2%)	
**Platelet count decreased**			0.69
Grade 1	4 (10%)	5 (12%)	
**Peripheral sensory neuropathy**			0.59
Grade 1	10 (24%)	11 (28%)	
Grade 2	1 (2%)	0 (0%)	
**Peripheral motor neuropathy**			0.16
Grade 1	1 (2%)	4 (10%)	
**Periorbital edema**			0.32
Grade 1	1 (2%)	0 (0%)	
**Paroxysmal atrial tachycardia**			0.31
Grade 1	0 (0%)	1 (2%)	
**Paresthesia**			0.32
Grade 1	1 (2%)	0 (0%)	
**Papulopustular rash**			0.57
Grade 1	2 (5%)	1 (2%)	
**Palpitations**			0.31

	Grade 1	0 (0%)	1 (2%)	
**Pain in extremity**				0.43
	Grade 1	3 (7%)	5 (12%)	
	Grade 2	0 (0%)	1 (2%)	
**Pain**				0.80
	Grade 1	5 (12%)	7 (18%)	
	Grade 2	1 (2%)	1 (2%)	
**Osteoporosis**				0.16
	Grade 1	2 (5%)	0 (0%)	
**Oral pain**				0.32
	Grade 1	1 (2%)	0 (0%)	
**Oral dysesthesia**				0.31
	Grade 1	0 (0%)	1 (2%)	
**Noncardiac chest pain**				0.20
	Grade 1	0 (0%)	2 (5%)	
	Grade 2	0 (0%)	1 (2%)	
**Neutrophil count decrease**				0.12
	Grade 1	9 (22%)	4 (10%)	
	Grade 2	16 (39%)	10 (25%)	
	Grade 3	10 (24%)	18 (45%)	
**Nervous system disorders**				0.31
	Grade 1	0 (0%)	1 (2%)	
**Neoplasms benign malignant**				0.32
	Grade 2	1 (2%)	0 (0%)	
**Neck pain**				0.32
	Grade 1	1 (2%)	0 (0%)	
**Nausea**				0.26
	Grade 1	16 (39%)	16 (40%)	
	Grade 2	5 (12%)	1 (2%)	
	Grade 3	1 (2%)	0 (0%)	
**Nasal congestion**				0.38
	Grade 1	2 (5%)	4 (10%)	
**Nail loss**				0.31
	Grade 1	0 (0%)	1 (2%)	
**Myalgia**				0.31
	Grade 1	5 (12%)	9 (22%)	
	Grade 2	1 (2%)	0 (0%)	
**Musculoskeletal and connective tissue disorders**				0.51
	Grade 1	1 (2%)	2 (5%)	
	Grade 2	1 (2%)	0 (0%)	
**Mucositis oral**				0.59
	Grade 1	7 (17%)	8 (20%)	
	Grade 2	1 (2%)	0 (0%)	

**Mitral valve disease**				0.59
	Grade 1	15 (37%)	15 (38%)	
	Grade 2	0 (0%)	1 (2%)	
**Memory impairment**				0.31
	Grade 1	0 (0%)	1 (2%)	
**Malaise**				0.35
	Grade 1	0 (0%)	1 (2%)	
	Grade 2	0 (0%)	1 (2%)	
**Lymphocyte count decrease**				0.84
	Grade 1	5 (12%)	6 (15%)	
	Grade 2	14 (34%)	9 (22%)	
	Grade 3	5 (12%)	5 (12%)	
	Grade 4	1 (2%)	1 (2%)	
**Lymphedema**				0.49
	Grade 1	6 (15%)	4 (10%)	
	Grade 2	1 (2%)	0 (0%)	
**Localized edema**				0.32
	Grade 1	1 (2%)	0 (0%)	
**Lip infection**				0.32
	Grade 2	1 (2%)	0 (0%)	
**Laryngeal inflammation**				0.31
	Grade 1	0 (0%)	1 (2%)	
**Irritability**				0.35
	Grade 1	0 (0%)	1 (2%)	
	Grade 2	0 (0%)	1 (2%)	
**Insomnia**				0.93
	Grade 1	5 (12%)	6 (15%)	
	Grade 2	1 (2%)	1 (2%)	
**Infections and infestations**				0.51
	Grade 1	1 (2%)	2 (5%)	
	Grade 2	1 (2%)	0 (0%)	
**Hypothyroidism**				1.00
	Grade 1	1 (2%)	1 (2%)	
	Grade 2	1 (2%)	1 (2%)	
**Hypotension**				0.32
	Grade 1	1 (2%)	0 (0%)	
**Hypophosphatemia**				0.43
	Grade 1	2 (5%)	3 (8%)	
	Grade 2	3 (7%)	6 (15%)	
	Grade 3	0 (0%)	1 (2%)	
**Hyponatremia**				0.91
	Grade 1	3 (7%)	2 (5%)	
	Grade 3	1 (2%)	1 (2%)	

**Hypomagnesemia**				0.17
	Grade 1	4 (10%)	1 (2%)	
**Hypokalemia**				0.97
	Grade 1	5 (12%)	5 (12%)	
**Hypoglycemia**				0.16
	Grade 1	2 (5%)	0 (0%)	
**Hypocalcemia**				1.00
	Grade 1	2 (5%)	2 (5%)	
	Grade 2	1 (2%)	1 (2%)	
**Hyperuricemia**				0.32
	Grade 1	1 (2%)	0 (0%)	
**Hypertension**				0.16
	Grade 1	2 (5%)	7 (18%)	
	Grade 2	2 (5%)	3 (8%)	
**Hypernatremia**				0.32
	Grade 1	1 (2%)	0 (0%)	
**Hyperkalemia**				0.32
	Grade 1	3 (7%)	1 (2%)	
**Hyperglycemia**				0.37
	Grade 1	9 (22%)	13 (32%)	
	Grade 2	1 (2%)	0 (0%)	
**Hypercalcemia**				0.99
	Grade 1	1 (2%)	1 (2%)	
**Hot flashes**				0.34
	Grade 1	13 (32%)	19 (48%)	
	Grade 2	1 (2%)	1 (2%)	
**Hiccups**				0.32
	Grade 1	1 (2%)	0 (0%)	
**Hemorrhoids**				0.31
	Grade 1	0 (0%)	1 (2%)	
**Hematuria**				0.31
	Grade 1	0 (0%)	1 (2%)	
**Hearing impaired**				0.32
	Grade 1	1 (2%)	0 (0%)	
**Headache**				0.82
	Grade 1	10 (24%)	10 (25%)	
	Grade 2	1 (2%)	2 (5%)	
**Generalized muscle weakness**				0.54
	Grade 1	1 (2%)	2 (5%)	
**General disorders and administration site condition**				0.59
	Grade 1	1 (2%)	1 (2%)	
	Grade 2	0 (0%)	1 (2%)	
**Gastrointestinal disorders**				0.35

	Grade 1	0 (0%)	1 (2%)	
	Grade 2	0 (0%)	1 (2%)	
**Gastroesophageal reflux disease**				0.16
	Grade 1	5 (12%)	1 (2%)	
	Grade 2	0 (0%)	1 (2%)	
**Gastritis**				0.31
	Grade 1	0 (0%)	1 (2%)	
**Gastric stenosis**				0.31
	Grade 1	0 (0%)	1 (2%)	
**Floaters**				0.57
	Grade 1	2 (5%)	1 (2%)	
**Flatulence**				0.31
	Grade 1	0 (0%)	1 (2%)	
**Fever**				0.30
	Grade 1	5 (12%)	2 (5%)	
	Grade 2	1 (2%)	0 (0%)	
**Febrile neutropenia**				0.31
	Grade 3	0 (0%)	1 (2%)	
**Fatigue**				0.19
	Grade 1	17 (41%)	23 (57%)	
	Grade 2	4 (10%)	1 (2%)	
	Grade 3	1 (2%)	3 (8%)	
**Fall**				0.12
	Grade 1	0 (0%)	2 (5%)	
	Grade 2	0 (0%)	2 (5%)	
**Esophageal pain**				0.32
	Grade 1	1 (2%)	0 (0%)	
**Electrocardiogram QTc prolonged**				0.51
	Grade 1	7 (17%)	8 (20%)	
	Grade 2	0 (0%)	1 (2%)	
	Grade 3	0 (0%)	1 (2%)	
**Ejection fraction decrease**				0.31
	Grade 2	0 (0%)	1 (2%)	
**Edema trunk**				0.31
	Grade 1	0 (0%)	1 (2%)	
**Edema limbs**				0.41
	Grade 1	4 (10%)	2 (5%)	
**Edema face**				0.54
	Grade 1	1 (2%)	2 (5%)	
**Dyspnea**				0.61
	Grade 1	3 (7%)	3 (8%)	
	Grade 2	1 (2%)	0 (0%)	
**Dysphagia**				0.32

	Grade 1	1 (2%)	0 (0%)	
**Dyspepsia**				0.91
	Grade 1	3 (7%)	2 (5%)	
	Grade 2	1 (2%)	1 (2%)	
**Dyspareunia**				0.31
	Grade 2	0 (0%)	1 (2%)	
**Dysgeusia**				0.16
	Grade 1	2 (5%)	0 (0%)	
**Dysesthesia**				0.32
	Grade 1	1 (2%)	0 (0%)	
**Duodenal hemorrhage**				0.31
	Grade 2	0 (0%)	1 (2%)	
**Dry skin**				0.57
	Grade 1	2 (5%)	1 (2%)	
**Dry mouth**				0.32
	Grade 1	1 (2%)	0 (0%)	
**Dry eye**				0.16
	Grade 1	1 (2%)	4 (10%)	
**Dizziness**				0.15
	Grade 1	6 (15%)	2 (5%)	
**Diarrhea**				0.71
	Grade 1	8 (20%)	9 (22%)	
	Grade 2	3 (7%)	1 (2%)	
	Grade 3	2 (5%)	1 (2%)	
**Dermatitis radiation**				0.57
	Grade 1	2 (5%)	1 (2%)	
**Depression**				0.37
	Grade 1	0 (0%)	1 (2%)	
	Grade 2	1 (2%)	0 (0%)	
**Dental caries**				0.31
	Grade 2	0 (0%)	1 (2%)	
**Dehydration**				0.31
	Grade 1	0 (0%)	1 (2%)	
**Creatinine increased**				0.78
	Grade 1	6 (15%)	5 (12%)	
**Cough**				0.61
	Grade 1	6 (15%)	6 (15%)	
	Grade 2	1 (2%)	0 (0%)	
**Constipation**				0.04
	Grade 1	17 (41%)	8 (20%)	
**Conjunctivitis**				0.16
	Grade 2	2 (5%)	0 (0%)	
**Confusion**				0.32

	Grade 1	1 (2%)	0 (0%)	
**Colitis**				0.32
	Grade 2	1 (2%)	0 (0%)	
**Cognitive disturbance**				0.22
	Grade 1	2 (5%)	0 (0%)	
	Grade 2	1 (2%)	0 (0%)	
**High cholesterol**				0.31
	Grade 1	0 (0%)	1 (2%)	
**Chills**				0.54
	Grade 1	1 (2%)	2 (5%)	
**Chest wall pain**				0.99
	Grade 1	1 (2%)	1 (2%)	
**Cardiac disorders**				0.32
	Grade 1	1 (2%)	0 (0%)	
**Bullous dermatitis**				0.99
	Grade 1	1 (2%)	1 (2%)	
**Bruising**				0.99
	Grade 1	1 (2%)	1 (2%)	
**Breast pain**				0.55
	Grade 1	3 (7%)	2 (5%)	
	Grade 2	1 (2%)	0 (0%)	
**Breast infection**				0.51
	Grade 2	1 (2%)	2 (5%)	
	Grade 3	1 (2%)	0 (0%)	
**Bone pain**				0.97
	Grade 1	3 (7%)	3 (8%)	
**Blurred vision**				0.51
	Grade 1	1 (2%)	2 (5%)	
	Grade 2	1 (2%)	0 (0%)	
**Blood bilirubin increased**				0.55
	Grade 1	3 (7%)	2 (5%)	
	Grade 2	0 (0%)	1 (2%)	
**Blood and lymphatic system disorders**				0.32
	Grade 2	1 (2%)	0 (0%)	
**Bloating**				0.99
	Grade 1	1 (2%)	1 (2%)	
**Back pain**				0.32
	Grade 1	6 (15%)	7 (18%)	
	Grade 2	0 (0%)	2 (5%)	
**Aspartate aminotransferase increase**				0.33
	Grade 1	9 (22%)	7 (18%)	
	Grade 2	3 (7%)	0 (0%)	
	Grade 3	1 (2%)	2 (5%)	

	Grade 4	0 (0%)	1 (2%)	
**Arthritis**				0.07
	Grade 1	0 (0%)	3 (8%)	
**Arthralgia**				0.22
	Grade 1	12 (29%)	12 (30%)	
	Grade 2	3 (7%)	0 (0%)	
**Aortic valve disease**				0.67
	Grade 1	3 (7%)	4 (10%)	
**Anxiety**				0.30
	Grade 1	4 (10%)	6 (15%)	
	Grade 2	2 (5%)	0 (0%)	
**Anorexia**				0.37
	Grade 1	6 (15%)	3 (8%)	
	Grade 2	0 (0%)	1 (2%)	
**Anemia**				0.51
	Grade 1	15 (37%)	17 (42%)	
	Grade 2	1 (2%)	0 (0%)	
	Grade 3	0 (0%)	1 (2%)	
**Alopecia**				0.55
	Grade 1	8 (20%)	9 (22%)	
	Grade 2	0 (0%)	1 (2%)	
**Allergic rhinitis**				0.57
	Grade 1	2 (5%)	1 (2%)	
**Allergic reaction**				0.31
	Grade 1	0 (0%)	1 (2%)	
**Alkaline phosphatase increase**				0.13
	Grade 1	6 (15%)	12 (30%)	
	Grade 2	0 (0%)	1 (2%)	
**Alanine aminotransferase increase**				0.24
	Grade 1	12 (29%)	9 (22%)	
	Grade 2	3 (7%)	0 (0%)	
	Grade 3	3 (7%)	1 (2%)	
	Grade 4	1 (2%)	2 (5%)	
**Abdominal pain**				0.22
	Grade 1	2 (5%)	5 (12%)	

Supplemental Table 2. Ribociclib discontinuation stratified by age, disease stage, menopausal status, and prior chemotherapy.

	Number of patients who discontinued early (%)	Number of patients who completed 12 cycles (%)	p-value
**Age**			0.089
< 50 years	5 (20%)	22 (39%)	
≥ 50 years	20 (80%)	34 (61%)	
**Disease stage**			0.16
I/II	20 (80%)	36 (64%)	
III	5 (20%)	20 (36%)	
**Menopausal status**			0.10
Pre- or perimenopausal	6 (24%)	24 (43%)	
Postmenopausal	19 (76%)	32 (57%)	
**Prior chemotherapy**			0.94
Adjuvant or neoadjuvant	19 (76%)	43 (77%)	
None	6 (24%)	13 (23%)	

Supplemental Table 3. Patient characteristics of patients who had ctDNA collected and successfully retrospectively analyzed for at least one timepoint (Successful ctDNA Sample) versus those who did not (Unsuccessful ctDNA Sample).

	Total (n = 81)	Successful ctDNA Sample (n = 42)	Unsuccessful ctDNA Sample (n = 39)	p-value
**Age at randomization, years**	54	52.9	55.1	0.12
**Age group ≤ 50**	27 (33%)	16 (38%)	11 (28%)	0.35
**Sex**				
Female	81 (100%)	42 (100%)	39 (100%)	
Male	0 (0%)	0 (0%)	0 (0%)	
**Menopausal status**				0.11
Pre- or perimenopausal	30 (37%)	19 (45%)	11 (28%)	
Postmenopausal	51 (63%)	23 (55%)	28 (72%)	
**Race**				0.19
Asian	4 (5%)	1 (2%)	3 (8%)	
Black or African American	3 (4%)	0 (0%)	3 (8%)	
More than one race	3 (4%)	1 (2%)	2 (5%)	
Other or unknown	7 (9%)	5 (12%)	2 (5%)	
White	64 (79%)	35 (83%)	29 (74%)	
**Ethnicity**				0.13
Hispanic or Latino	5 (6%)	4 (10%)	1 (3%)	
Non-Hispanic	63 (78%)	29 (69%)	34 (87%)	
Unknown	13 (16%)	9 (21%)	4 (10%)	

**Disease stage**				0.28
I	11 (14%)	8 (19%)	3 (8%)	
II	45 (56%)	23 (55%)	22 (56%)	
III	25 (31%)	11 (26%)	14 (36%)	
**Histological grade**				0.93
1	11 (14%)	6 (14%)	5 (13%)	
2	46 (57%)	23 (55%)	23 (59%)	
3	24 (30%)	13 (31%)	11 (28%)	
**Estrogen receptor-positive**	81 (100%)	42 (100%)	39 (100%)	
**Progesterone receptor status**				0.47
Low positive	1 (1%)	1 (2%)	0 (0%)	
Positive	71 (88%)	37 (88%)	34 (87%)	
Negative	8 (10%)	3 (7%)	5 (13%)	
Unknown	1 (1%)	1 (2%)	0 (0%)	
**Surgery**				0.74
Bilateral mastectomy	26 (32%)	13 (31%)	13 (33%)	
Unilateral mastectomy	29 (36%)	16 (38%)	13 (33%)	
Lumpectomy or conservation	25 (31%)	13 (31%)	12 (31%)	
None	1 (1%)	0 (0%)	1 (3%)	
**Radiotherapy**				0.90
Adjuvant	71 (88%)	37 (88%)	34 (87%)	
None	10 (12%)	5 (12%)	5 (13%)	
**Chemotherapy (general)**				0.55
Adjuvant or neoadjuvant	62 (77%)	31 (74%)	31 (79%)	
None	19 (23%)	11 (26%)	8 (21%)	
**Anthracycline therapy**				0.32
Yes	46 (57%)	24 (57%)	22 (56%)	
No	33 (41%)	18 (43%)	15 (38%)	
Unknown	2 (2%)	0 (0%)	2 (5%)	
**Taxane therapy**				0.18
Yes	61 (75%)	29 (69%)	32 (82%)	
No	20 (25%)	13 (31%)	7 (18%)	

Supplementary Methods

Patients must have completed definitive surgery for their breast cancer, could have received (neo)adjuvant chemotherapy, and could have received adjuvant radiotherapy. Patients with QTc $\geq$ 470ms, Eastern Cooperative Oncology Group performance score of ≥ 2, known breast cancer metastases, uncontrolled intercurrent illness, and/or prior exposure to CDK 4/6, PI3K, and/or mTOR inhibitors were excluded. Prior tamoxifen use was permitted. Patients on tamoxifen were switched to an AI prior to enrollment. The study protocol and all amendments were approved by the institutional review board prior to implementation.

Patients were treated for one year or until meeting discontinuation criteria. Criteria for discontinuation included radiographic or symptomatic disease recurrence, unacceptable toxicity, intercurrent illness prevented further administration of treatment, inability or unwillingness to comply with the oral medication regimen and/or documentation requirements, withdrawal of informed consent, or changes in the participant's condition rendering the participant unacceptable for further treatment in the judgment of the treating investigator.

In-person visits occurred biweekly for the first two months (Cycle 1 Days 1, 15; Cycle 2 Days 1, 15) and bimonthly for the subsequent 10 months (every even cycle). AEs were assessed at each patient visit: in-person (Cycle 1, 2, and every subsequent even cycle) and by phone (every odd cycle, except for Cycle 1). In the event of AEs, treatment was interrupted or delayed and resumed only if protocol-defined criteria were met. Dose reductions and delays were permitted as pre-specified by the protocol.
